# Optimisation of the internal structure of ceramic membranes for electricity production in urine-fed microbial fuel cells

**DOI:** 10.1016/j.jpowsour.2020.227741

**Published:** 2020-03-01

**Authors:** M.J. Salar-García, I. Ieropoulos

**Affiliations:** Bristol BioEnergy Centre, Bristol Robotics Laboratory, T-Block, UWE Coldharbour Lane, Bristol BS16 1QY, UK

**Keywords:** Microbial fuel cells, Bioenergy, Ceramic membranes, Porosity, Pore size, Bulk resistance, Urine

## Abstract

The need to find a feasible alternative to commercial membranes for microbial fuel cells (MFCs) poses an important challenge for the practical implementation of this technology. This work aims to analyse the influence of the internal structure of low-cost terracotta clay-based membranes on the behaviour of MFCs. To this purpose, 9 different combinations of temperature and time were used to prepare 27 MFC separators. The results show that the temperature has a significant effect on both porosity and pore size distribution, whereas the ramp time do not show a significant influence on these parameters. It was observed that kilning temperatures higher than 1030 °C dramatically reduce the porosity of the samples, reaching a minimum value of 16.85%, whereas the pore size increases as the temperature also increases. Among the membranes with similar porosities, those with a medium pore size distribution exhibited the lowest bulk resistance allowing MFCs to reach the highest power output (94.67 μW cm^−2^). These results demonstrate the importance of not only the porosity but also the pore size distribution of the separator in terms of MFC performance and longevity, which for these experiments was for 90 days.

## Highlights

• Temperatures higher than 1030 °C dramatically reduces the porosity of the membranes.
• The ramp time showed a significant effect on neither porosity nor pore size.
• Medium pore size allowed MFC to reach the highest power output (94.67 μW cm^−2^).
• The maximum power output was obtained with membranes kilned at 1030 °C and 7 h.
• The urine-fed systems were working for 90 days and showed good long-term stability.

## 1 Introduction

In recent years, there has been a growing concern about the environmental impact of humankind behaviour and therefore, the interest in developing clean technologies has exponentially increased. Microbial fuel cells (MFCs) emerged to address two of the most important current environmental challenges: i) the need for finding a renewable alternative to fossil fuels and ii) water scarcity in many regions of the world [1,2].

MFCs are bioelectrochemical systems capable of producing bioenergy and treat wastewater simultaneously by using bacterial metabolism. The structure of an MFC consists of an anodic chamber, where bacteria oxidised the organic matter contained in a broad range of substrates, and a cathodic chamber where the reduction of an oxidant takes place, which is usually oxygen. Both chambers are physically separated by a selective separator or membrane [[3], [4], [5]]. The suitability of different substrates for bioenergy production in MFCs has been investigated over the years. The substrates range from simple compounds, such as acetate, to more complex feedstock, such as wastewater or urine. Among the different types of substrates, complex types of feedstock have been reported to be more promising for MFC practical applications, whereas simple materials are more suitable for laboratory scale and preliminary work [6,7]. In the last decade, great efforts have been made for optimising the design of these devices as well as maximising the energy harvesting. The MFC structure, along with the materials employed to build them up, have a significant impact on their performance, the cost of the overall system and therefore, on their practical application [[8], [9], [10]]. So far, carbon based-materials are widely used to elaborate the electrodes due to their low cost and good conductivity and biocompatibility. However, the cathode still needs the presence of a catalyst to accelerate the sluggish cathodic reaction. Catalysts containing platinum group metals are the most widely used in MFCs due to their high catalytic activity in a wide pH domain. However, their integration in commercial systems is mainly hindered by their high cost and tendency to get poisoned under the presence of certain pollutants, which limit their longevity. To overcome these drawbacks and facilitate the commercialisation of MFCs, the use of platinum group metal free materials or metal-free carbonaceous materials to catalyse the cathodic reaction is gaining importance [[11], [12]].

Regarding the separator, commercial polymer-based membranes such as Nafion or Ultrex are the most widely used due to their good performance. However, their high cost makes their use infeasible in scaled-up systems for practical applications [13]. To overcome the drawbacks of using commercial membranes, alternative materials have been investigated as MFC separators, with ceramics being some of the most promising due to their low cost and natural availability. Earthenware, terracotta or clayware are some of the most commonly used because of their robustness for long-term processes and low maintenance requirements, which facilitates their use in commercial applications [14,15]. One of the emerging applications of MFCs is in remote locations or developing countries for treating wastewater and simultaneously producing bioenergy. According to this approach, environmentally friendly and cost-effective materials, such as ceramic materials, have gained much attention in recent years [[16], [17], [18], [19]]. In addition to their natural availability and low cost, another benefit of ceramic membranes is that their internal structure can be finely tuned by varying both the raw material and the kilning procedure [20,21]. The variation of ceramic properties such as porosity, pore size distribution or internal resistance of the structure might affect the flux of ions through the membranes and therefore the power performance of MFCs [22]. Kilning temperature and time have a crucial impact on the final ceramic structure, which can go from a high degree of porosity to full vitrification very quickly. The presence of uniform fine pores in the structure of ceramic membranes may ensure efficient separation of electrolyte while allowing diffusion of material without any mass flow limitations [23].

However, despite the fact that use of ceramic materials in different research fields has significantly increased due to the multitude of benefits, published reports regarding the effect of the ceramic internal structure on the MFC performance, are still limited. For these reasons, this work aims to control the internal structure of terracotta-clay membranes by varying the kilning method in order to maximise the power performance of MFCs. To this extent, the effect of porosity, pore size distribution and bulk resistance on the MFC performance has been investigated in-depth.

## 2 Materials and methods

### 2.1 Microbial fuel cell set-up

The air-breathing single-chamber MFC set-up consisted of a cubical assembly made of acrylic material with an effective volume of 11.75 mL. The air-facing cathodes were made of a blend of activated carbon and polytetrafluoroethylene (PTFE) (186 ± 7 mg cm^−2^) [24] pressed over a piece of stainless steel mesh (12.25 cm^2^) whereas the anodes consisted of carbon veil (20 g m^−2^, PRF composites, Dorset. the UK) coated with activated carbon (5 mg cm^−2^). As a membrane, flat pieces of terracotta clay (3.5 × 3.5 cm^2^) were cut and kilned at three different ramp times – meaning the length of time taken to reach the target temperature – and subsequently held at that temperature for 3 min; the three different temperatures were: 860, 1030 and 1140 °C and the 3 different ramp times were: 5, 7 and 9 h (see Table 1). These temperature and time parameters were selected according to previous experiments, which determined the minimum conditions to get well-performing membranes, whereas the maxima were determined by the limiting conditions of the apparatus. After kilning, the final thickness of the membranes was 3 mm. The number of temperature/time combinations studied was 9 and the samples were assessed in triplicate, with a total number of 27 MFC units for the whole experiment.

**Table 1:** Summary of the 9 combinations of temperature and time used to kiln the ceramic membranes.

	Temperature (°C)	Ramp time (h)
Temperature/Time combinations	860	5
		7
		9
	1030	5
		7
		9
	1140	5
		7
		9

### 2.2 Microbial fuel cell inoculation and operation

The air-breathing MFCs were inoculated with a mixture of sludge and neat human urine (1:1 v/v) in batch mode. This solution was replenished with a fresh mixture, every 24 h for 4 days. The systems were subsequently continuously fed with urine at a continuous feed flow rate of 0.06 mL min^−1^ and the external resistive loading was adjusted during the maturing period after which it was kept constant at 900 Ω. MFC voltage was continuously monitored by using a multichannel Agilent recorder data logger (LXI 34972A data acquisition/Switch unit) for 90 days.

### 2.3 Electrochemical measurements

The polarisation of MFCs using the different terracotta membranes was performed using a potentiostat (μAutoLab III/FRA2, Metrohm, The Netherlands) by linear sweep voltammetry (LSV) from open-circuit voltage (OCV) to 0.05 mV at a scan rate of 0.25 mV s^−1^. The MFCs were left in OCV for at least 2 h before performing the measurements to allow the stabilisation of the OCV. The two-electrode technique was used with the anode connected to the counter electrode, the cathode connected to the working electrode and reference channel short-circuited with the counter electrode channel. Polarisation curves were obtained by plotting the cell voltage *versus* current (V *vs.* I) whereas power curves were obtained by plotting power *versus* current (P *vs*. I). Power was obtained by multiplying voltage and current. The obtained power was normalised as power density with respect to the area of the cathode in contact with the membrane.

### 2.4 Porosity and pore size distribution analysis

The internal structure of the terracotta clay membranes was analysed in terms of total porosity and pore size distribution by using mercury intrusion porosimetry (Poremaster-60 GT, Quantachrome Instrument, United Kingdom). This device was equipped with dual high-pressure transducers to enhance accuracy across the entire analytical range and also included two built-in automated low-pressure ports for filling of penetrometers and intrusion/extrusion measurements from vacuum to 60.000 psi. In this case, the samples were dried at 60 °C overnight and then placed under vacuum before starting the measurements. The intrusion measurements were performed in the following range of pressure 6.548 KPa to 408167.656 KPa, whereas the extrusion measurements were performed between 405003.562 KPa and 138.412 KPa. Mercury porosimetry analysis is a widely used technique for analysing the pore size distribution as well as the porosity of different nature materials [25].

### 2.5 Impedance spectroscopy (IES)

IES technique was used to determine the ionic conductivity of the terracotta clay membranes by using the μAutoLab III with a frequency response analyser FRA2 (Metrohm, The Netherlands). The range of frequency selected to perform the measurements was the following: 100 kHz to 10 mHz, at AC amplitude of 10 mV in a two electrodes configuration where the reference electrode was short-circuited to the counter electrode. The conductivity of each sample was determined by extracting the bulk resistance (R_b_) by the intersection of the semicircle with the Z’ axis in the Nyquist plot [[26], [27], [28]]. Then, the ionic conductivity of the different membranes was calculated by using the following equation:

[1] $$\sigma =\frac{\text{L}}{A\times R_{b}}$$

Where σ is the ionic conductivity (S.cm^−1^), L (cm) the thickness of the membrane, A (cm^2^) the contact area between the electrodes and the ceramic membrane and R_b_ (Ω) is extracted from the Nyquist plot, as previously commented [28].

The IES measurements were performed by using the same MFC set-up that was employed for running the experimental tests but using buffer pH 9 as electrolyte and two thin pieces of carbon veil as electrodes. According to this configuration, both the R_b_ and the conductivity are mainly related to each type of membrane analysed. These parameters have been reported to be crucial for well-performing MFCs [28].

## 3 Results and discussion

### 3.1 Membrane characterisation

The total porosity of the different membranes elaborated was determined by using mercury intrusion porosimetry (see Table 2). The results show that there are no significant changes in the total porosity of the samples kilned at 860 °C and 1030 °C regardless of the time. In the first case, this parameter ranges from 27.67% for the faster ramp of time to 26.65% for the longest one. Similar results were observed for membranes kilned at 1030 °C where the porosity varies from 25.37% for 5 h of a ramp time to 26.12% for 9 h. On the contrary, more significant changes were observed in membranes kilned at 1140 °C, where the porosity reduces as the ramp time increases. For kilning temperatures higher than 1030 °C the results show a dramatic decline in porosity, reaching a minimum value of 16.84%. These results show that among the different conditions studied, temperatures higher than 1030 °C strongly reduce the porosity whereas the ramp time does not show a significant effect on this parameter for low/medium kilning temperatures [29,30].

**Table 2:** Total porosity of the different ceramic membranes tested.

	Total porosity (%)		
Ramp time (h)	860 °C	1030 °C	1140 °C
5	27.67	25.37	19.99
7	25.8	26.08	16.84
9	26.65	26.12	17.85

**Fig. 1:**
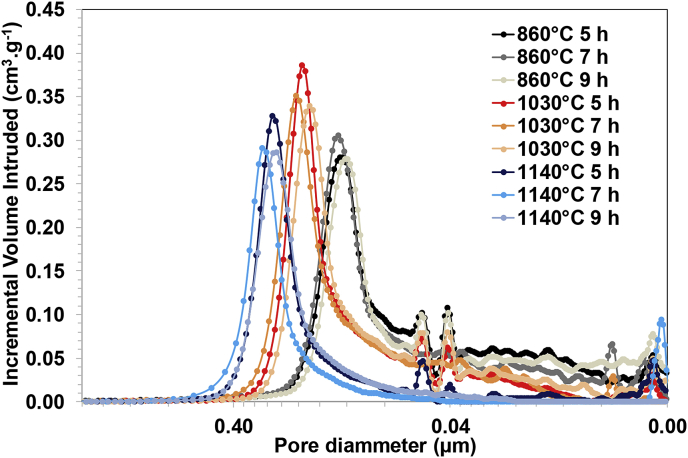
Pore size distribution of the different ceramic membranes analysed: A) Kilned at 860 °C, B) Kilned at 1030 °C, C) Kilned at 1140 °C and D) Overlapped spectra.

With regard to the pore size distribution, Fig. 1 shows the results of this parameter obtained from the mercury intrusion porosimetry measurements. As can be observed, the ramp time does not affect significantly the pore size distribution of the membranes. However, the pore size increases as the kilning temperature also increases, regardless of time. For instance, comparing the pore size distribution for membranes kilned at different temperatures and 7 h ramp time, the results show that when the kilning temperature is 860 °C, 94.05% of the pores are between 0.0039 and 0.2635 μm. By increasing the temperature up to 1030 °C, 92.24% of the pores are between 0.0132 and 0.3894 μm. Finally, for the highest temperature tested, it was observed that 87.38% of the membrane pores range between 0.0189 and 0.5080 μm. According to these results, the pore size distribution of membranes kilned at 1140 °C is two times larger than for membranes kilned at 860 °C. Similar behaviour is observed for the rest of ramp times evaluated (See Fig. 1). It can also be seen that the smallest pores remain in the membrane structure for medium/low temperatures regardless of the ramp time used. However, for the highest temperature, the smallest pores disappear as the ramp time increases, which may be due to their coalescence (see Fig. 1); it is actually a common characteristic that kilning at higher temperatures results in denser materials. These results possibly suggest the absence of carbonates in the raw clay, which results in a reduction in porosity and increase in the pore size as the kilning temperature increases because of the coalescence of smaller pores [[29], [30], [31], [32]].

**Fig. 2:**
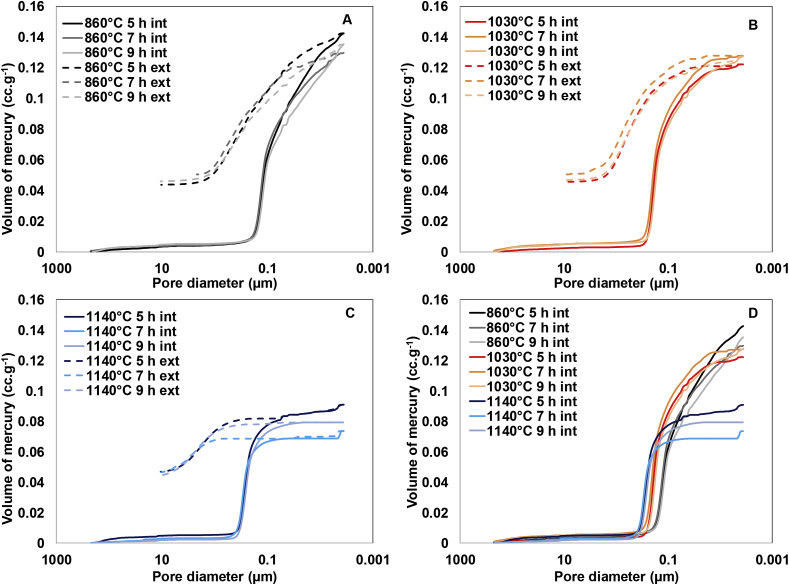
Volume of mercury intruded (solid line) and extruded (dashed line) in the porous surface of the different ceramic membranes tested: A) Kilned at 860 °C, B) Kilned at 1030 °C, C) Kilned at 1140 °C and D) Overlapped volume of mercury intruded of the different membranes.

Fig. 2A, B and C display the cumulative volume of the mercury intruded and extruded in the porous surface of the different membranes elaborated. As can be seen in Fig. 2D, the volume of mercury intruded decreases as the kilning temperature increases, being higher in the case of membranes kilned at 860 °C and lower for the membranes made at 1140 °C. These results could be attributed to the small pores removal whereas those remained become larger, which are in line with the pore size distribution previously discussed. The plots also show that the extrusion curve does not follow the intrusion curve. This means that the intrusion/extrusion cycle is not close when the process goes back to the initial pressure, which indicates that some mercury is entrapped in the sample pore space. The different volume of mercury intruding and extruding might be related to the presence of either an intricate pore network or the pores show bottleneck effects [31,33,34].

**Fig. 3:**
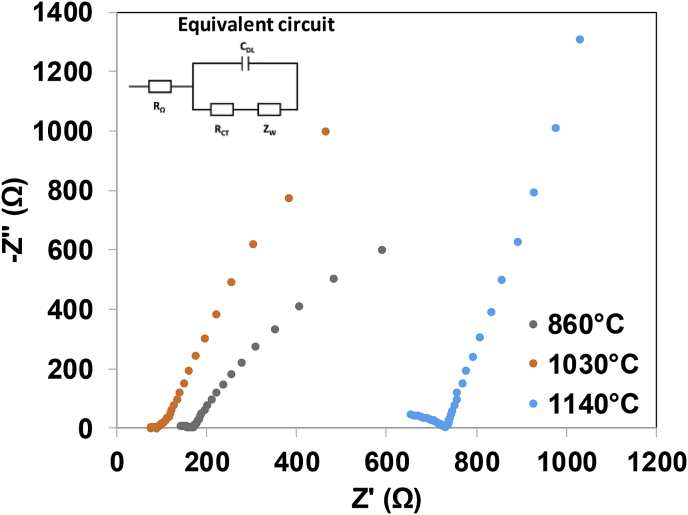
Nyquist plot obtained from the IES of the membranes kilned at 860 °C, 1030 °C and 1140 °C at a ramp time of 7 h.

Fig. 3 shows the Nyquist plot from the IES performed with the different ceramic membranes elaborated at a time ramp of 7 h and the equivalent circuit of the system. As can be seen, there is a noticeable difference in the ohmic resistance recorded for the different samples, with increasing value for those membranes with lower porosity in Table 2.

**Fig. 4:**
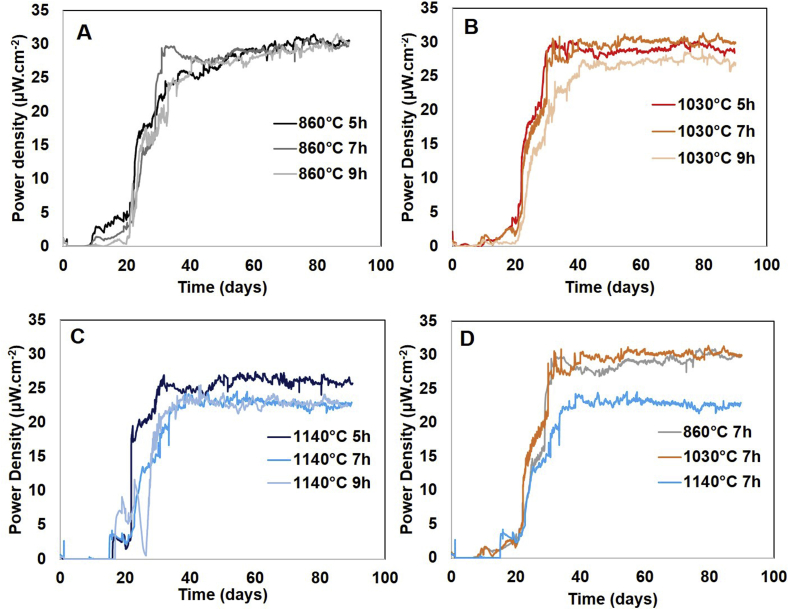
Long-term performance of the MFCs working with the different ceramic membranes prepared. A) Kilned at 860 °C, B) Kilned at 1030 °C, C) Kilned at 1140 °C and D) Overlapped results of the membranes kilned at all temperatures studied and 7 h of ramp time.

The Nyquist plots obtained from the three membranes analysed exhibit the same shape, which comprises a semicircle in the high-frequency region and an inclined line in the low-frequency region caused by the diffusion species into the electrode material. This type of response is named as Warburg-type behaviour, and it is widely observed in fuel cells [35]. A complete semicircle is commonly obtained in a Nyquist plot at high-frequency region whereas an incomplete semicircle might be observed at lower frequency regions. In our case, the three samples show an incomplete semicircle. These results might be related to the presence of intricate pore in the ceramic structure of the samples kilned at the highest temperature. To determine the R_b_, the data were forecasted until reaching the intersection of the semicircle with the Z’ axis (see Fig. 4). From an electrical point of view, Fig. 4 also shows the equivalent circuit of the Nyquist plot obtained where R_Ω_ is the ohmic resistance, C_DL_ is the double layer capacitance, Zw is the Warburg impedance and R_CT_ is the charge transfer resistance of the cathode [36].

Regarding the ionic conductivity of each membrane elaborated at a ramp time of 7 h, this parameter was calculated by using equation (1) and is shown in Table 3, along with the R_b_ values. According to the data obtained, there is a correlation between the R_b_ and therefore, the ionic conductivity and the porosity of the samples. The porosity of the membranes elaborated at a ramp time of 7 h (Table 2) shows the following trend according to the kilning temperature: 1030 °C > 860 °C ≫ 1140 °C, which is inversely proportional to R_b_. The results show that despite membranes kilned at 1140 °C exhibit larger pore size than those elaborated at a lower temperature, their structure is less porous and the pores are more intricate so the R_b_ is significantly higher than the rest of samples. As expected, a more dense membrane, will pose a higher ohmic resistance [35]. The highest conductivity (3.5 × 10^−4^ S cm^−1^) was obtained for the medium kilning temperature (1030 °C).

**Table 3:** Bulk resistance and ionic conductivity of each of the different membranes elaborated at a ramp time of 7 h.

Kilning temperature (°C)	R_b_ (Ω)	σ (S.cm^−1^)
860	114.2	2.1 × 10^−4^
1030	70.6	3.5 × 10^−4^
1140	497.2	0.49 × 10^−4^

### 3.2 Microbial fuel cell performance

Long-term stability of MFCs is a crucial factor to consider regarding their practical implementation. In this work, the MFCs were running for 90 days (3 months) in continuous mode. Once the systems were matured, the voltage remained stable for more than 60 days, regardless of the type of membrane used. Fig. 4 depicts the evolution of the average voltage over time for each membrane assessed in triplicate. During the operating time, once the steady-state was reached (around day 30). The effect of the ramp time during the kilning process on the power performance of MFC was not significant for membranes prepared at 860 °C and 1030 °C (see Fig. 4A and B). However, in the case of membranes kilned at the highest temperature the power output decreases as the ramp of time increases, being maximum in the case of 5 h of ramp time (see Fig. 4C). These results are in line with the minimal changes observed in both porosity and pore size distribution when the ramp time is modified for kilning temperatures equal or below 1030 °C. However, comparing the different groups of temperatures, membranes kilned at low/medium temperature allow MFCs to reach higher values of power than those kilned at 1140 °C. MFCs working with membranes kilned at 1030 °C and 7 h ramp time were able to reach a stable power density up to 30.07 μW cm^−2^ (368.36 μW), 24% higher than systems working with membranes kilned at 1140 °C during the same time (see Fig. 4D). These results might be related to the low porosity of the membranes elaborated at high temperature, which increases the internal resistance of the material and therefore, reduces the MFC performance [35].

It is well known from literature that ceramic materials show many characteristics, which make them suitable for being used as a separator in MFCs [37,38]. A cuboid air-breathing MFC set-up, similar to that used in the present work, was well-performing during 90 days reaching a stable power output of 41.2 ± 0.02 μW cm^−2^ when the cathode contained iron-streptomycin derived catalyst [39]. Recently, Theodosiou et al. (2019) investigated the use of different kind of materials as MFC separators and compared them with terracotta clay in air-breathing MFCs set-up. Their results showed that MFCs working with terracotta membranes outperformed those working with a commercial cation exchange membrane during 70 days operation in batch mode [40]. On the other hand, fine fire clay (modified and unmodified) membranes were kilned at different temperatures and their performance as MFC separators was compared [35]. In this case, all the systems exhibited stable power performance for 60 days. Among the unmodified fire fine clay membranes, those kilned at the lowest temperature (1150 °C) allowed the MFCs to reach the highest power output. A similar behaviour was observed in the case of the modified membranes being those kilned at the lowest temperature the most efficient in terms of power performance.

**Fig. 5:**
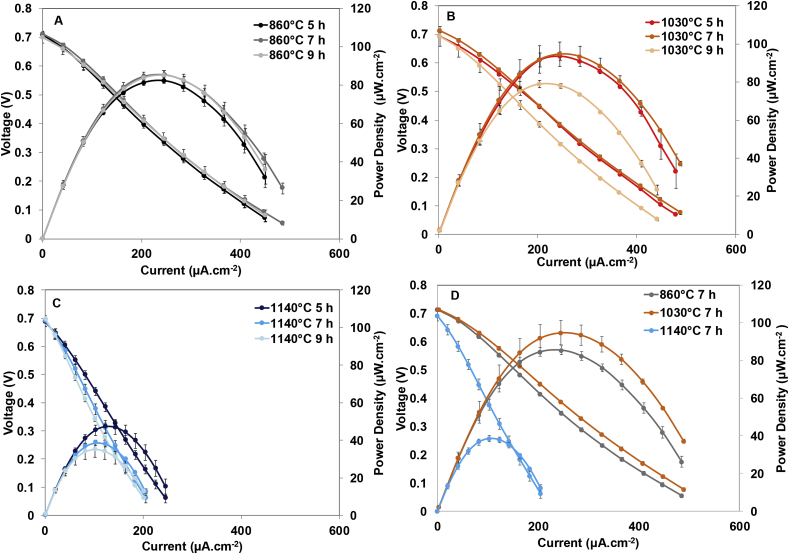
Power and Polarisation curves of MFCs working with the different membranes elaborated: A) Kilned at 860 °C, B) Kilned at 1030 °C, C) Kilned at 1140 °C and E) Overlapped results of the membranes kilned at all temperatures studied and 7 h of ramp of time.

Fig. 5 shows the electrochemical performance of the overall cells when they reached a steady-state. According to the long-term power output previously discussed, the overall polarisation curves showed that the ramp time does not significantly affect this parameter for membranes prepared at low/medium temperature. However, in the case of the membranes kilned at 1140 °C the maximum power output by the MFCs substantially decreases as the ramp time increases, being minimum in the case of the longest kilning process. Fig. 5D shows the power curves of the membranes elaborated at 860 °C, 1030 °C and 1140 °C and 7 h of ramp time. As can be seen, the power output by MFCs working with the membranes prepared at 1030 °C is higher than the rest of systems, reaching up to 94.67 μW cm^−2^ (1.16 mW) of power output, 9.82% higher than membranes kilned at 860 °C and up to 59.21% higher than membranes prepared at 1140 °C.

According to these results, the kilning temperature and therefore the internal structure of the membranes significantly affect the power performance of the MFCs. In this case, temperatures higher than 1030 °C decreased the power output because of the reduction in porosity coupled to the increase in R_b_. These results are in line with those reported by Merino-Jimenez et al. (2019) who also observed an increase in the R_b_ of fine fire clay membranes when the temperature of the kilning cycle increased [35]. It has also been reported that terracotta clay membranes kilned at 1070 °C and 7 h of ramp time allow MFCs to reach up to 104.5 μW cm^−2^ when iron-based materials are used as a catalyst [39], which is in the same order of magnitude as the power output generated by membranes kilned at 1030 °C and 7 h of ramp time in the present work.

**Fig. 6:**
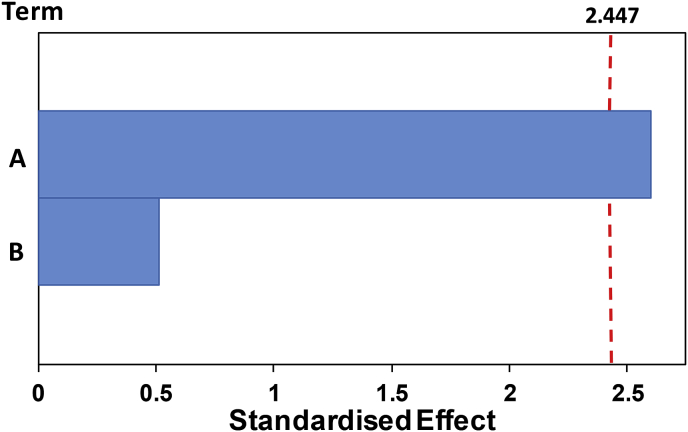
Pareto chart for power (α = 0.05) where the temperature is defined as term A whereas the ramp time is defined as term B.

Table 4 shows the analysis of variance (ANOVA) in power for the input variables studied, ramp time and temperature (Minitab 19 ©). The results show that among the conditions studied, only temperature has a significant effect with 95% confidence (p < 0.05). Pareto chart, which depicts the standardised effect with p = 0.05 reaches the same conclusion as that of the variance analysis (see Fig. 6). The bar length belongs to the absolute standardised value. Only the bar related to term A (temperature) surpasses the reference line (2.447), meaning that it is the only effect that is statistically significant.

**Table 4:** Analysis of variance (ANOVA) in power.

Source	DF	Adj SS	Adj MS	F-Value	P-Value
Regression	2	2477.95	1238.97	3.55	0.096
Ramp Time	1	92.71	92.71	0.27	0.625
Temperature	1	2385.24	2385.24	6.84	0.040
Error	6	2092.54	348.76		
Total	8	4570.49

**Fig. 7:**
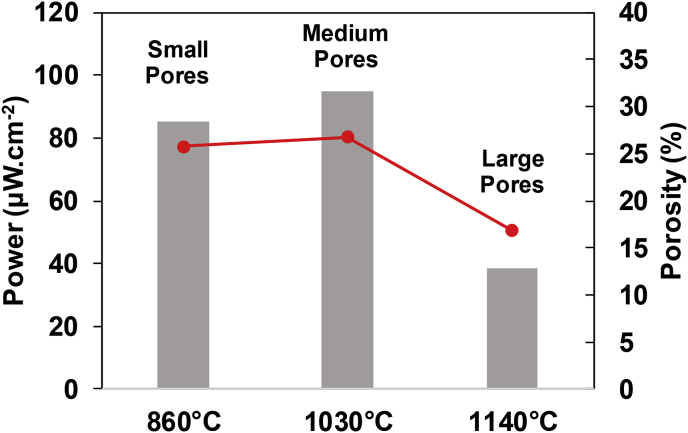
Relationship between temperature, porosity, pore size and power output for samples prepared at 7 h of ramp time.

From these results, the relationship between the kilning temperature, the porosity and pore size of the ceramic membranes and the power output by MFCs can be derived and is shown in Fig. 7. As can be observed, the dramatic reduction in porosity caused by the highest kilning temperature shows a direct effect on power performance. As previously discussed, membranes with similar porosities (25.8 and 26.8%) allow the system to reach similar values of power output, whereas lower values of porosity (16.84%) negatively affect the MFC performance. The pore size, grouped into small, medium and large according to the pore size distribution analysis, also affects the power output. The results show that large pores coupled to low porosity of the ceramic membrane do not align with high performance. According to the results obtained, medium pores improve the performance of the system compared to small pores, for similar values of porosity. This pore size might be ideal for avoiding losses of anolyte without being blocked, as well as reducing the diffusion of oxygen from the cathode side. These results confirm the importance of controlling the kilning conditions of terracotta clay membranes in order to optimise their internal structure and therefore maximise the power output by MFCs.

## 4 Conclusions

The use of ceramic membranes as a low-cost alternative to commercial membranes in MFCs brings numerous advantages but it is crucial to optimise their structure in order to improve the power performance of these devices. In this work, 3 temperatures and 3 ramps times were combined with a total number of 9 different membranes assessed in triplicate (27 MFCs). Internal properties of the membranes such as porosity, pore size distribution or bulk resistance have been determined and their effect on the MFC power performance evaluated. The results show that the power density, as well as the porosity of the samples, is very similar for kilning temperatures between 860 °C and 1030 °C. However, the use of higher temperatures dramatically reduces the porosity of the membranes, reaching a minimum value of 16.85%. Regarding the pore size, it was observed an increase as the kilning temperature also increases, maybe due to the coalescence of smaller pores. By contrast, the influence of the time was not significant on both parameters for low/medium temperatures. Among the membranes with similar porosities, those with a medium pore size distribution exhibited the lowest bulk resistance allowing MFCs to reach the highest power output (94.67 μW cm^−2^, 1.16 mW). Regarding the ionic conductivity of the membranes, this parameter is closely related with MFC power output, since both parameters follow the same trend according to the kilning temperature: 1030 °C > 860 °C > 1140 °C. Despite membranes elaborated at 1140 °C have larger pores than the rest of samples, their number is more reduced and they are more intricate, which increases the bulk resistance of the membrane and therefore, the performance of MFCs. The power output was increased by 59.21% only by reducing the kilning temperature from 1140 °C to 1030 °C. The statistical analysis reported that in this case, only the kilning temperature significantly affects the power performance of MFCs. The results showed that this parameter directly affects the porosity, pore size and R_b_ of terracotta clay membranes, which demonstrates the importance of controlling the internal structure of this type of materials in order to maximise the power output by MFCs. Moreover, the systems were working for 90 days and showed good longevity, which reinforces the use of these natural membranes in MFCs and therefore, facilitating their practical applications.

## Declaration of competing interests

The authors declare that they have no known competing financial interests or personal relationships that could have appeared to influence the work reported in this paper.
